# *Bocavirus* [bō-kə-vī-rəs]

**DOI:** 10.3201/eid1303.06407

**Published:** 2007-04

Genus in the family *Parvoviridae*. Previously identified members of this genus are pathogens of bovines and canines. A parvovirus of human origin was recently discovered and called human bocavirus because it is closely related to bovine parvovirus and canine minute virus. Human bocavirus is associated with respiratory tract infections, particularly in infants and young children.

